# Synergy-based Functional Electrical Stimulation for gait rehabilitation in chronic stroke: a pilot study

**DOI:** 10.3389/fresc.2026.1847719

**Published:** 2026-09-09

**Authors:** Jackson Taylor Levine, Xin S. Yu, Rebecca Muñoz, Alaina Fiorenza, Irina Djuraskovic, J. D. Peiffer, Emilia Ambrosini, Simona Ferrante, Elliot J. Roth, Jozsef Laczko, Ronald James Cotton, Alessandra Pedrocchi, Jose L. Pons

**Affiliations:** 1 McCormick School of Engineering, Northwestern University, Evanston, IL, United States; 2 Shirley Ryan AbilityLab, Chicago, IL, United States; 3 Doctor of Physical Therapy Program, College of Health Sciences, University of Memphis, Jackson, TN, United States; 4 Department of Physical Medicine and Rehabilitation, Northwestern University, Chicago, IL, United States; 5 Department of Neuroscience, Northwestern University, Evanston, IL, United States; 6 Department of Electronics, Politecnico Di Milano, Milan, Italy; 7 Faculty of Information Technology and Bionics, Pazmany Peter Catholic University, Budapest, Hungary

**Keywords:** Functional Electrical Stimulation, gait, muscle synergies, rehabilitation, stroke

## Abstract

**Introduction:**

Chronic stroke gait disorders involve impaired motor coordination. While high-intensity gait training (HIGT) is supported by current clinical practice guidelines, and Functional Electrical Stimulation (FES) to tibialis anterior addresses foot drop, extending FES to multiple muscles may improve functional outcomes.

**Methods:**

Using a FES neural sleeve capable of recording muscle activity and joint angles, we tested preliminary efficacy and feasibility of a personalized multichannel FES (MFES) intervention based on the individual’s motor coordination impairment paired with HIGT. Fourteen individuals with chronic stroke were randomly assigned to either HIGT or MFES + HIGT for six weeks. Gait speed, endurance, gait biomechanics, and muscle synergies were assessed at baseline, midpoint, post-training, and one-month follow-up.

**Results:**

Despite the small sample, only the MFES + HIGT group demonstrated significant endurance gains from baseline to post-training and follow-up, while both groups improved walking speed, impaired limb step length, and muscle synergy similarity to normative data. Feasibility was evaluated by measuring setup time (therapist putting device on participant and setting stimulation parameters) and collecting feedback from participants and four therapists. System setup time plateaued at 4.53 min by the ninth session. Both participants and therapists rated the intervention highly feasible, acceptable, and usable. Adherence was high in both groups, with only one dropout in the HIGT group due to preexisting medical conditions.

**Discussion:**

This pilot study demonstrates the preliminary effectiveness of synergy-based MFES in improving walking endurance and confirms its feasibility for integration into chronic stroke gait rehabilitation, supporting the need for larger-scale trials to validate clinical efficacy and identify responders.

**Clinical Trial Registration:**

https://clinicaltrials.gov/study/NCT06099444, identifier NCT0609944.

## 1 Introduction

Gait impairment is one of the most common and debilitating consequences of stroke, affecting up to 80% of individuals with stroke and significantly limiting mobility and independence (1). Post-stroke gait is often slower, more fatiguing, and marked by poor coordination and compensatory strategies (2–4). Although high-intensity gait training (HIGT) is supported by current clinical practice guidelines (5) and has shown substantial benefits during the subacute phase of recovery (6, 7), recovery potential with current approaches is still modest during the chronic phase and may be enhanced through novel approaches like neuromodulation (8). Despite the availability of novel assistive technologies such as robotic exoskeletons and electrical stimulation devices, their clinical use remains limited due to time to use, complexity, and lack of integration into existing therapy workflows (9). As a result, HIGT emphasizes gait speed and target heart rate but may not adequately address individualized motor coordination deficits that persist in chronic stroke.

When paired with task-oriented movement intent, Functional Electrical Stimulation (FES) can induce peripheral benefits (10) and enhance central nervous system neuroplasticity (11–15) by providing timed muscle activation and sensory feedback and helping to reshape neural control through repetitive training (16, 17). It is most commonly used to treat foot drop through stimulation of the tibialis anterior (TA) during swing phase, reducing compensatory patterns such as circumduction and hip hiking (2, 18). However, single-muscle stimulation has demonstrated limited and heterogeneous benefits over standard of care, particularly in individuals with chronic stroke (19–21). Extending FES to multiple muscles has shown some moderate improvements, such as improved endurance, gait velocity, and gait symmetry with the addition of the gluteus medius (GM) (22, 23) or reduced energy expenditure with the addition of the gastrocnemius (GAS) in the FastFES paradigm (24, 25).

The complexity of post-stroke gait, which often includes impaired intermuscular coordination and the heterogeneous effects of FES training, underscore the need for broader and more individualized stimulation strategies—potentially involving a larger array of muscles and proper identification of responders to different therapies. One prominent indicator of this impairment is the reduction in number of muscle synergies and the presence of abnormal coordination patterns, which can be quantified through muscle synergy analysis of electromyography (EMG) signals (26). Muscle synergies represent the coordinated activation patterns produced by the nervous system to control movement, are consistent and reproducible across walking speeds (27, 28), and are linked to biomechanical functions of gait: weight acceptance, knee flexion, propulsion, foot clearance (26, 29, 30). After stroke, these synergies often become disrupted, leading to inappropriate timing of muscle activations and abnormal coactivation of muscles. Notably, individuals with impaired synergies (e.g., reduced number of synergies, reduced similarity of synergies to neurologically intact individuals) exhibit more impaired gait (e.g., slower speed, shorter step length, asymmetries) (27). Those who demonstrate improved locomotor function following rehabilitation often show muscle synergy patterns that more closely resemble those of neurologically intact individuals (31–33), suggesting rehabilitation can restore more physiologically appropriate muscle coordination, thereby improving gait function and biomechanics. These findings also highlight the potential of muscle synergies to serve both as biomarkers and therapeutic targets for stroke recovery.

To better target these coordination deficits, we propose a personalized rehabilitation intervention that delivers multichannel FES (MFES) based on an individual's impaired muscle synergies precisely timed during HIGT to reduce gait impairment in individuals with chronic stroke. This approach provides feedback to the major leg muscles (i.e., plantarflexors, dorsiflexors, knee flexors, knee extensors, hip abductors) according to the individual's specific neural coordination deficits, quantified through muscle synergy analysis at baseline, and has also been demonstrated in the upper limb (34–38). Our preliminary work (39) and that of colleagues (40) using traditional manually placed electrodes demonstrated proof of concept of synergy-based MFES in two individuals each with chronic stroke during a four-week gait training intervention, and found improved walking speed and similarity of synergies to neurologically intact individuals immediately after training. However, this has not been investigated in comparison to standard of care. Additionally, development of wireless, wearable leg sleeves that provide MFES [e.g., Cionic Neural Sleeve (41, 42), Kurage] can reduce setup time and simplify complexity of use by standardizing electrode placement and supportive user interfaces, therefore facilitating their integration into clinical workflows.

In this pilot study, we aimed to evaluate preliminary efficacy, identify potential responders/non-responders, and assess clinical feasibility of a six-week synergy-based MFES combined with HIGT compared to HIGT alone in individuals with chronic stroke. Given the small sample size and unrestrictive inclusion criteria, we hypothesized that only the synergy-based MFES group would exhibit statistically significant and clinically meaningful improvements in gait speed and endurance. We recognize that this study is not powered to identify between group differences, and the objective is to demonstrate early efficacy and feasibility to support a large scale randomized controlled trial. Furthermore, we hypothesized that therapists would quickly become proficient in setting up and applying personalized synergy-based MFES and be able to integrate it efficiently within typical therapy timelines.

## 2 Materials and methods

### 2.1 Device

**Figure 1:**
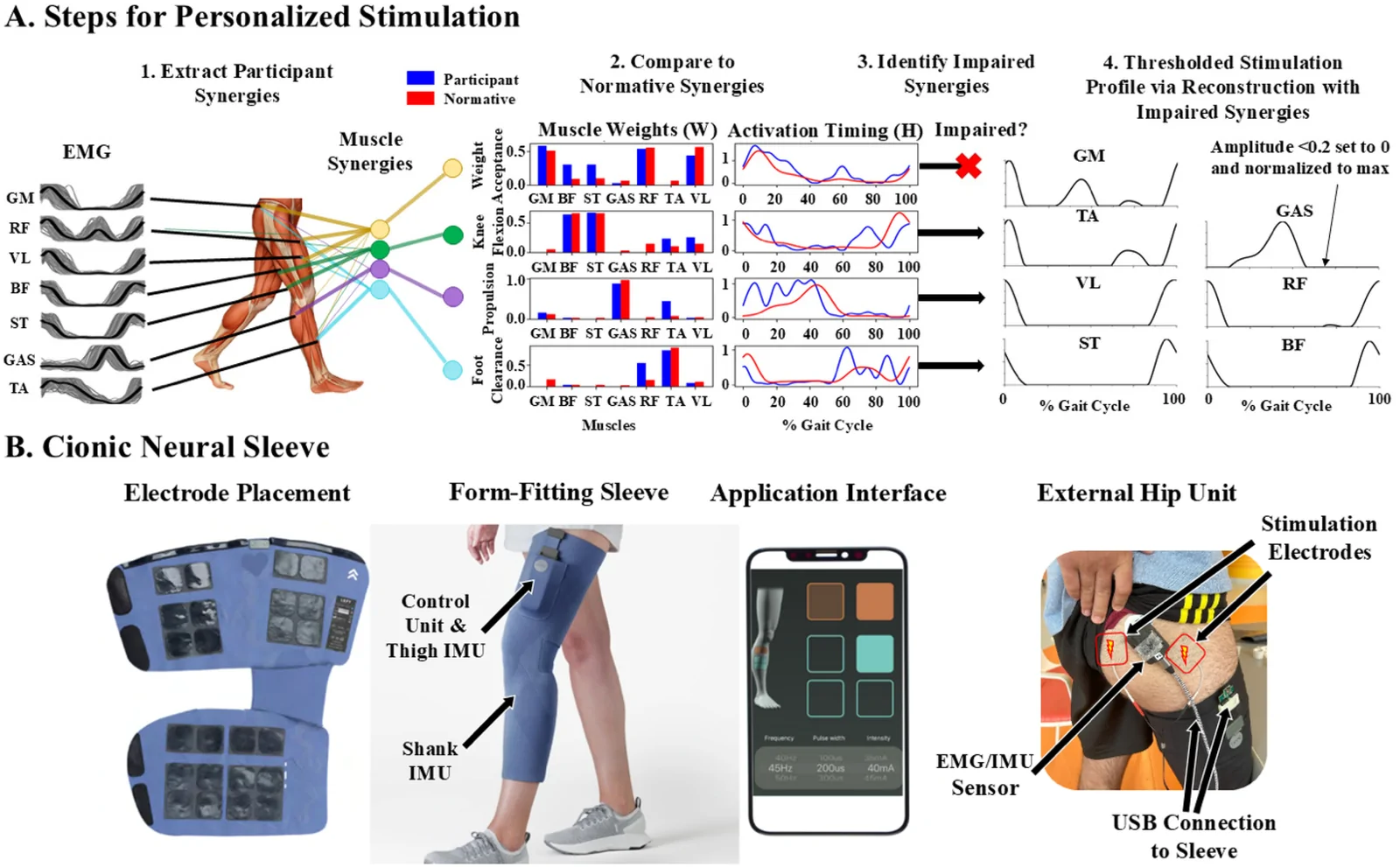
Personalized synergy-based stimulation. A) Steps for identifying impaired synergies, computing personalized stimulation based on impaired synergies, and precisely timed stimulation profiles normalized to maximum and thresholded to remove any values below 0.2. B) Cionic Neural Sleeve with electrode placement and the application interface, which was used to set and store stimulation parameters. Placement of the custom external hip unit is displayed, with labels for the stimulation electrodes and sensor for recording electromyography (EMG) and inertial measurement unit (IMU) signals. GM, Gluteus Medius; GAS, Gastrocnemius; TA, Tibialis Anterior; VL, Vastus Lateralis; RF, Rectus Femoris; BF, Biceps Femoris; ST, Semitendinosus.

This study used a modified version of the Cionic Neural Sleeve (Figure 1B), an FDA-approved device [510(k) number K21362] intended to provide ankle dorsiflexion and/or plantarflexion in adult individuals with foot drop and/or to assist knee flexion or extension in adult individuals with muscle weakness related to upper motor neuron disease/injury (e.g., stroke, damage to pathways to the spinal cord) (41, 42). The sleeve consists of electrodes that both record EMG and provide monophasic stimulation for key lower limb muscles (tibialis anterior (TA), gastrocnemius (GAS), rectus femoris (RF), vastus lateralis (VL), semitendinosus (ST), and biceps femoris (BF)) with two embedded inertial measurement units (IMUs) on the shank and thigh.

A custom external hip unit was developed by Cionic to target the hip abductor, gluteus medius (GM), which was also equipped with EMG and IMU sensors (depicted in right panel of Figure 1B). The hip unit was worn separately from the neural sleeve and positioned over the lateral hip using two bipolar surface EMG electrodes to enable stimulation and sensing of the GM. Although physically separate, the hip unit was connected directly to the neural sleeve control unit through a wired interface and did not operate as an independent device. The shared controller provided power, stimulation control, and data acquisition, allowing EMG, IMU, and stimulation signals from both the sleeve and hip unit to be synchronized within a common timing framework and recorded simultaneously. The hip unit used the same stimulation hardware and electrode design as the neural sleeve. GM is involved in pelvic stabilization, which is often impaired post-stroke and can result in abnormal weight bearing and weight shifting (23). EMG and IMU signals were recorded from the Cionic Neural Sleeve and sampled at 2000Hz and 100 Hz, respectively.

### 2.2 Sample

**Figure 2:**
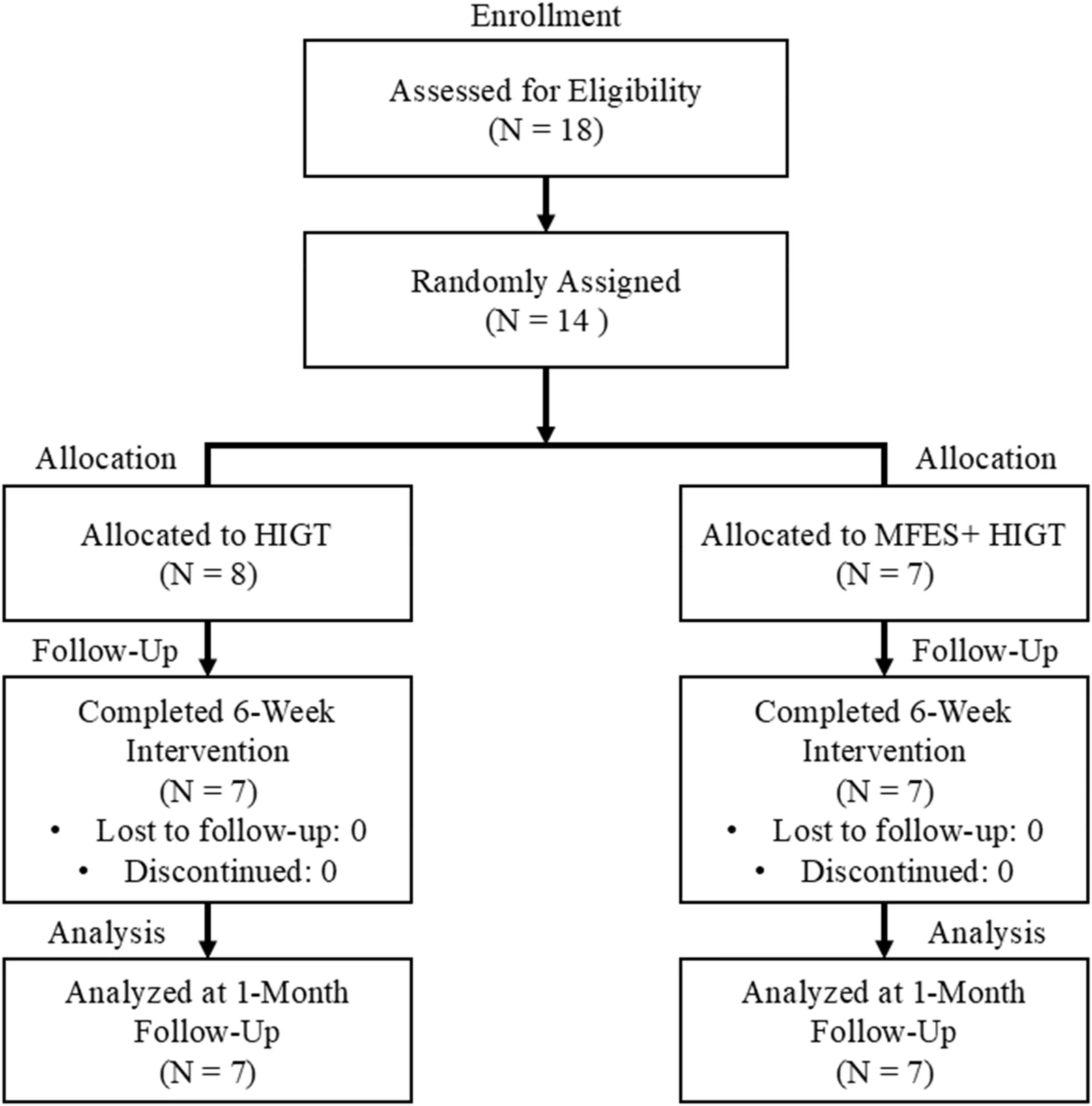
CONSORT flow diagram of participant progress through the pilot randomized controlled trial of a high-intensity gait training (HIGT) versus HIGT combined with muscle synergy-based multichannel functional electrical stimulation (MFES) in individuals with chronic stroke.

Fourteen individuals with chronic, unilateral stroke (>6 months) and reduced but independent ambulation (for at least 10 m) were recruited. Exclusion criteria included multiple strokes, other neurological or orthopedic impairments, contraindications to FES, concurrent lower limb studies and unable to understand and give informed consent. Participants were randomly assigned to one of two groups: (1) HIGT (*N* = 7) or (2) HIGT in combination with synergy-based MFES (MFES + HIGT) (*N* = 7) (Figure 2). All participants provided informed consent at the initial visit and then completed a baseline assessment. Fourteen neurologically intact individuals were recruited to establish normative synergies for comparison with stroke synergies.

To assess clinical feasibility, four physical therapists were recruited to deliver MFES + HIGT protocol (these therapists did not perform any assessments). All therapists provided informed consent and completed two hours of training on both the rehabilitation protocol and use of the Cionic Neural Sleeve prior to being paired with an individual post-stroke for the six-week intervention. On average, therapists had 4.5 years of experience in post-stroke gait rehabilitation and were familiar with single-channel FES for foot drop (primarily using the Bioness device to stimulate the TA). The study protocol was approved by the Northwestern University Institutional Review Board (STU00218244, date of approval: 8/17/2023, clinical trial number: NCT0609944, date of first enrollment: starting 6/24/2024) and conducted in accordance with the Declaration of Helsinki.

### 2.3 Intervention

Participants trained three times weekly for six weeks (eighteen sessions), walking on a Woodway clinical treadmill with a harness (no weight support) per clinical guidelines (5). Each session included a five-minute warm-up and cool down at self-selected velocity (SSV, determined at baseline assessment) and 20 min of HIGT with or without synergy-based MFES. Therapists adjusted treadmill speed and incline to challenge participants to reach 70%–85% of their maximum heart rate (HR) (5) and Borg Rating of Perceived Exertion (RPE) between 15 and 17 (43). HR was measured using a Polar OH1 sensor over wrist extensors or biceps (44, 45).

For participants in the MFES + HIGT group, therapists put the sleeve and custom hip unit on the impaired limb of the participant. Stimulation amplitudes (15–100 mA) were set per muscle via the mobile tablet application (Figure 1B) at the discretion of the clinician such that the amplitude produced a visible or palpable contraction of the target muscle while maintaining participant comfort. Frequency (30 Hz) and pulse width (300 µs) were fixed. Motor-level personalized stimulation was applied during the 20-min training segment. Throughout training, participants and clinicians communicated regarding participant perception of stimulation levels, which were adjusted on the mobile tablet application if stimulation became uncomfortable or insufficient. Stimulation amplitudes were saved after the first training and updated for each training, if needed. Setup time was recorded to assess feasibility.

### 2.4 Personalized stimulation control

Personalized stimulation was designed after the baseline assessment and used for every training session, as described in our previous work validating the concept (Figure 1A) (39). A more in-depth visualization of the steps for personalization are described in Supplementary Figure S1.

#### 2.4.1 Participant EMG processing and muscle synergies

Participant EMG (*M_Pt_*) was recorded at baseline during treadmill walking at SSV (determined during baseline 10MWT). Signals were bandpass filtered (3rd order, 40–400 Hz cutoff, Butterworth filter), rectified, and low pass filtered (3rd order, 5 Hz cutoff, Butterworth). They were segmented into gait cycles, interpolated from 0 to 100% of the gait cycle, and normalized to peak-to-peak amplitude (Figure 1A Step 1). Normative muscle synergy weights (W) and activation profiles (H) were computed as the average of fourteen neurologically intact individuals (age: 46.9 ± 17.0) walking at ten speeds ranging from 0.45 m/s to 1.8 m/s for one minute each using nonnegative matrix factorization (NNMF) and a variance accounted for threshold of 0.9 to define the number of synergies (four synergies were extracted on average). Following extraction, synergies were matched across all subject–speed trials by identifying the combination of synergy weight vectors that maximized cosine similarity between trials. After alignment, the corresponding synergy weights (W) and activation profiles (H) were averaged across all matched trials to obtain the normative synergies (46). Consistent with the approach proposed by Ferrante et al. (2016), participant-specific synergy weights (*W_Pt_*) and activation profiles (*H_Pt_­*) were reconstructed within the normative synergy framework prior to comparison. That is, participant-specific synergy weights (*W_Pt_*) and activation profiles (*H_Pt_­*) were computed using an iterative non-negative matrix reconstruction approach where normative weights (*W_Norm_*) were fixed to reconstruct the participant activation profiles and the normative activation profiles (*H_Norm_*) were fixed to reconstruct the participant weights. This approach ensures that each participant synergy corresponds directly to a specific normative synergy, avoiding ambiguity associated with independently extracted NNMF solutions. Equations are provided below with full algorithmic details are in Ferrante et al (39).

$$W_{Pt}^{i+1}=W_{Pt}^{i}\frac{\left(M_{Pt},\;\cdot H_{Norm}^{T}\right)}{\left(W_{Pt}^{i}\cdot H_{Norm}\cdot H_{Norm}^{T}\right)}$$ (1)

$$H_{Pt}^{i+1}=H_{Pt}^{i}\frac{\left(W_{Norm}^{T}\cdot M_{Pt},\right)}{\left(W_{Norm}^{T}\cdot \;W_{Norm}\cdot H_{Pt}^{i}\right)}$$ (2)

#### 2.4.2 Identification of impaired participant synergies

Impairment metrics were calculated using the reconstructed participant-specific synergy weights and activation profiles obtained from Equations (1) and (2). Participant synergies were compared to average normative synergies, and the degree of deviation was quantified using cosine similarity of weights W, circular cross correlation coefficient of activation profiles H, and circular cross correlation time lag of activation profiles H (39). The time lag was computed as $Tlag=1-\left|\frac{lag}{100}\right|$, where lag represents a percentage of the gait cycle (value between −50% and 50%). The mean and standard deviation for all three metrics across muscle synergies of neurologically intact individuals were computed in comparison to the average normative synergies. For each participant synergy, comparisons to normative synergies were computed, and a synergy was classified as impaired if any of the three metrics deviated by more than two standard deviations from the normative (Figure 1A Step 3).

#### 2.4.3 Personalized stimulation profiles

Only impaired synergies were used to drive the personalized stimulation profile development. The normative weights and activation profiles of synergies that were deemed impaired for each participant were multiplied to define a continuous stimulation profile for a complete gait cycle for each muscle. The stimulation profile was normalized between zero and one. The stimulation profiles were designed to follow the temporal activation patterns of the corresponding muscle synergies. For visualization, the EMG activation profiles and stimulation profiles were independently normalized; therefore, their 0–1 scales are not directly comparable and should not be interpreted as equivalent amplitudes.

To prevent continuous stimulation throughout the gait cycle and minimize fatigue, only stimulation values exceeding 0.2 were included in the stimulation profile. These values were then scaled to the maximum stimulation amplitude set by the therapist through the interface (Figure 1A Step 4). Stimulation profiles were triggered using the IMU sensors integrated into the shank and thigh segments of the sleeve. Gait cycle duration was continuously updated using an exponential moving average based on the previous ten gait cycles, with peak shank angle serving as an indicator of heel strike. To provide phase-specific stimulation timing, the gait cycle was segmented in real time using characteristic peaks and zero-crossings in the sagittal-plane shank and thigh IMU signals. These IMU events were identified from gait data collected in neurologically intact individuals and were used as reference markers for gait phase transitions. The identified gait events with their corresponding time points throughout the gait cycle (0%–100%) were as follows: (1) peak shank angle (0/100% gait cycle), corresponding to heel strike; (2) negative zero-crossing of the shank angular velocity (∼28%), corresponding to mid-stance; (3) minimum thigh angle (∼57%), corresponding to toe-off; (4) positive zero-crossing of the thigh angular velocity (∼68%), corresponding to midswing; (5) minimum shank angle (∼73%), corresponding to late swing; and (6) positive zero-crossing of the shank angular velocity (∼91%), corresponding to terminal swing, immediately preceding the next heel strike. For each muscle, the onset of the stimulation profile was associated with the gait event occurring closest to the desired stimulation onset. For each ramp up or down in intensity, a fixed delay (% gait cycle) between the detected gait event and stimulation onset was then applied so that stimulation occurred at the intended point within the gait cycle. The fixed delay was multiplied by the duration of the gait cycle, which was updated with an exponential average. The IMU events and triggering/delay process is visualized in Supplementary Figure 1.

### 2.5 Assessment visits and outcome measures

#### 2.5.1 Assessment visit

Assessments were performed at baseline, midpoint, post (immediately after completing training), and follow-up (one month after completing the training). The baseline assessment was completed one week prior to the beginning of training. During this week, the participant wore a step-counting device (ActiGraph, USA) (47–49) to track average daily number of steps taken as a measure of participation. This was repeated immediately following completion of all training sessions. At each assessment, participants completed the 10-Meter Walk Test (10MWT) at both SSV and fast velocity (FV) and the 6-Minute Walk Test (6MWT). During the 10MWT at SSV, participants were recorded using a markerless motion capture system (50). Participants were permitted to use the same assistive device for all assessment points (e.g., cane or walker), but ankle-foot orthoses were not allowed to assess intrinsic ankle motor control impairments. Three levels of ambulation are reported based on the self-selected walking speed during 10MWT at baseline: household (<0.4 m/s), limited community (0.4–0.8 m/s), and community (>0.8 m/s) (51). Participants then donned the Neural Sleeve and external hip sensor on the impaired limb. Following a two-minute warm-up, participants walked on a treadmill for two minutes at their SSV while IMU angular data and EMG from the same muscles used for stimulation were recorded.

#### 2.5.2 Gait biomechanics

Markerless motion capture data taken during 10MWT SSV was processed according to Cotton et al. (50, 52). Videos were collected from multiple calibrated and synchronized cameras. Participant tracking using EasyMocap (53) and 2D joint location estimation using MeTRAbs-ACAE (54) was facilitated using PosePipe (55). We used an end-to-end reconstruction approach to jointly learn the body scale parameters and joint angles of a differentiable biomechanical model most consistent with video observations (52). From these biomechanical reconstructions, we extracted gait event timings (56, 57) and calculated spatiotemporal measures: impaired limb step length, step width, double support percent, and impaired limb single support percent. Kinematic analysis focused on joint movements and phases specific to stroke impairment on the impaired limb: peak ankle dorsiflexion during swing (foot clearance), peak hip abduction during swing (circumductory gait compensation), peak hip extension during stance (propulsion), peak knee extension during stance (stance stability and hyperextension), and peak knee flexion during swing.

#### 2.5.3 Muscle synergies

Cosine similarity (as a measure of similarity of the muscles involved in a synergy) and circular cross correlation (as a measure of similarity of synergy activation timing) were assessed for the impaired limb compared to normative synergies. Both measures vary from 0 to 1, with 0 representing the lowest similarity and 1 representing the greatest similarity between individuals with stroke and neurologically intact individuals. These measures were computed for each synergy, and the average among synergies impaired at baseline was reported. Additionally, the number of synergies extracted from NNMF where variance accounted for plateaued was reported (58).

### 2.6 Clinical feasibility

To obtain participant feedback on the intervention, all participants completed the Patient Perceived Usefulness Questionnaire, with the prompt “With respect to your walking ability, how would you describe yourself now compared to before starting your rehabilitation program?” at the follow-up assessment ranging from −5 (Much Worse) to 5 (Completely Recovered) (59). Adverse reactions were recorded.

To assess clinical feasibility, the time required for a clinician to don the sleeve and set and adjust stimulation parameters was recorded for each session for a single participant per therapist, defined as the first participant they trained, in order to characterize changes in performance associated with therapist learning over the course of training. Timing was complete after the sleeve was donned and stimulation parameters were set for all muscles. Time required to remove the device was not measured. To assess the ability of therapists to challenge their participants to achieve the requirements of HIGT, mean HR, peak HR, percentage of time spent in the target HR zone, and RPE were evaluated for each session. Therapists also completed the Therapist Perceived Usefulness Questionnaire, with the prompt “With respect to the rehabilitation program used for this patient, how likely would you be to integrate this into your clinical practice?”, ranging from −5 (Very Unlikely) to 5 (Very Likely) (59). Additionally, therapists completed two more surveys: Brooke System Usability Scale (60) and Weiner Acceptability, Appropriateness and Feasibility of Intervention Measure (61). All therapist forms were collected after completing at least six training sessions.

### 2.7 Statistics

SPSS 26.0.0.1 was used for statistical analysis. To compare group demographics, a linear mixed model was used with Group (HIGT, MFES + HIGT) as a fixed effect and Participant as a random effect. HR and RPE were compared using a linear mixed model with Session Number and Group as fixed effects and Participant and Therapist as random effects.

For efficacy measures, a linear mixed model was used with Group and Visit (pre, mid, post, follow-up) as fixed effects and Participant as a random effect. Assumptions of linearity, independence, homoscedasticity, normality of residuals, and absence of multicollinearity were verified and transformations applied where needed according to Shapiro–Wilk's test (62, 63). If residuals remained non-normal after transformation, data were analyzed using generalized linear mixed models (peak ankle dorsiflexion of the impaired limb during swing, peak knee extension of the impaired limb during stance, average circular cross-correlation and cosine similarity of impaired synergies). *Post-hoc* comparisons were made with Bonferroni corrections and *α* = 0.05.

Due to the inability of household ambulators (i.e., SSV < 0.4 m/s) to reach target HR, an exploratory sub-group analysis was conducted on the primary outcomes—clinical assessments—excluding participants that were categorized as household ambulators at baseline (*N* = 2 from MFES + HIGT, *N* = 1 from HIGT) using a linear mixed model with Group and Visit as fixed effects with Participant as a random effect.

A linear plateau model was applied in Rstudio 2025.05.0 to determine the inflection point at which setup time plateaued. Setup durations from 57 total sessions conducted by four physical therapists (PT1: 13 sessions, PT2: 15 sessions, PT3: 15 sessions, PT4: 14 sessions) were analyzed.

## 3 Results

### 3.1 Participant demographics

Of eighteen screened, fourteen participants (three females per group) completed training. Three did not pass screening while one in the HIGT group dropped out of the study due to pre-existing medical issues before mid-point and therefore was not included in the analysis. Demographics are displayed in Table 1. There were no differences between groups in age (HIGT: 60.6 ± 10.7, MFES + HIGT: 57.7 ± 10.2, *p* = 0.331), years since stroke (*p* = 0.703), baseline 10MWT SSV (*p* = 0.625), nor baseline 6MWT (*p* = 0.462).

**Table 1:** Participant demographics.

ID	Group	Sex	Hemi Side	Years Since Stroke	Stroke Type	Assist Device	Pre SSV (m/s)	Pre 6MWT (m)	Baseline Ambulatory Group
1	HIGT	Female	Right	21	Hem	n/a	0.49	208	Limited
2	HIGT	Female	Right	5	Isch	n/a	0.69	250	Limited
3	HIGT	Male	Left	6	Isch	n/a	0.91	371	Community
4	HIGT	Male	Right	4	Isch	Cane	0.58	243	Limited
5	HIGT	Male	Right	15	Unk	Roller Walker	0.73	201	Limited
6	HIGT	Female	Left	2	Isch	n/a	1.01	366	Community
7	HIGT	Male	Left	6	Hem	Cane	0.23	79	Household
7	Mean ± Std	4M/3F	4R/ 3L	8.4 ± 6.9			0.66 ± 0.26	245.4 ± 101.2	1 House, 4 Limited, 2 Community
8	MFES + HIGT	Male	Left	3	Hem	n/a	1.35	502	Community
9	MFES + HIGT	Female	Left	8	Hem	Cane	0.4	133	Limited
10	MFES + HIGT	Male	Right	12	Isch	n/a	0.81	394	Community
11	MFES + HIGT	Male	Left	10	Hem	Cane	0.15	47	Household
12	MFES + HIGT	Male	Left	2	Hem	Cane	0.41	164	Limited
13	MFES + HIGT	Female	Left	15	Isch	Cane	0.11	43	Household
14	MFES + HIGT	Female	Left	3	Isch	n/a	0.75	58	Limited
7	Mean ± Std	4M/3F	1R/ 6L	7.6 ± 5.1			0.57 ± 0.44	191.6 ± 183.6	2 House, 3 Limited, 2 Community

Hem, Hemorrhagic; Isch, Ischemic; Unk, Unknown; SSV, Self-Selected Velocity; 6MWT, 6 Minute Walk Test.

### 3.2 Training intensity

Training and cardiovascular intensity results demonstrate that on average both groups maintained target HR intensities for 50% of each session, consistent with other HIGT studies (7). In addition, average RPE was 14.1 ± 3.2 for HIGT and 14.9 ± 2.9 for MFES + HIGT, indicating that participants perceived the intensity of the intervention close to “Hard’. The percentage of time spent in the target heart rate zone was 72.6 ± 30.2% and 49.2 ± 37.6% for HIGT and MFES + HIGT, respectively. The three household ambulators (2 in MFES + HIGT, 1 in HIGT) spent on average 22.9 ± 14.3% in the target heart rate zone, and when excluded from the analysis, participants in the HIGT group averaged 78.1 ± 21.1% and MFES + HIGT group averaged 62.9 ± 29.6%. This limitation motivated investigation with exclusion of more severely impaired participants (i.e., household ambulators) that did not achieve training goals. Despite the lower percentage of time spent in the target heart rate zone, these participants averaged above 13 for their RPE rating. Regardless, there was no main effect of group, session, nor group*session interaction for mean HR, peak HR, percentage of time in HR zone, nor mean RPE. *P*-values are reported along with mean and standard deviations for all metrics considering all participants and all sessions (see Table 2). There were no adverse reactions in either group to training.

**Table 2:** Training and cardiovascular intensity details.

Metric	HIGT	MFES + HIGT	*P*-value
Mean HR (% Max)	75.0% (69.0%—79.0%)	67.0% (65.0%—81.0%)	0.31
Peak HR (% Max)	84.0% (75.0%—85.0%)	74.0% (73.0%—89.0%)	0.28
Percent Time in HR Zone (%)	74.0% (42.0%—96.2%)	40.5% (21.8%—93.0%)	0.17
Mean RPE	14.6 (12.0–16.0)	16.0 (13.2–16.6)	0.65

Median and interquartile range are reported. A linear mixed effects model with fixed effects of group and session and a random effect of subject was conducted, and the *p*-value of the group effect is reported. There was no effect of session nor a group*session interaction. HIGT, High-Intensity Gait Training; MFES, Multichannel Functional Electrical Stimulation; HR, Heart Rate; RPE, Borg Rating of Perceived Exertion (Range = 6–20).

### 3.3 Training efficacy

No differences were identified between groups at baseline for clinical, biomechanical, nor synergy metrics (all *p* > 0.05). Data of all participants are displayed in Supplementary Table S1, and exclusion of household ambulators in Supplementary Table S2.

#### 3.3.1 Clinical assessments

**Figure 3:**
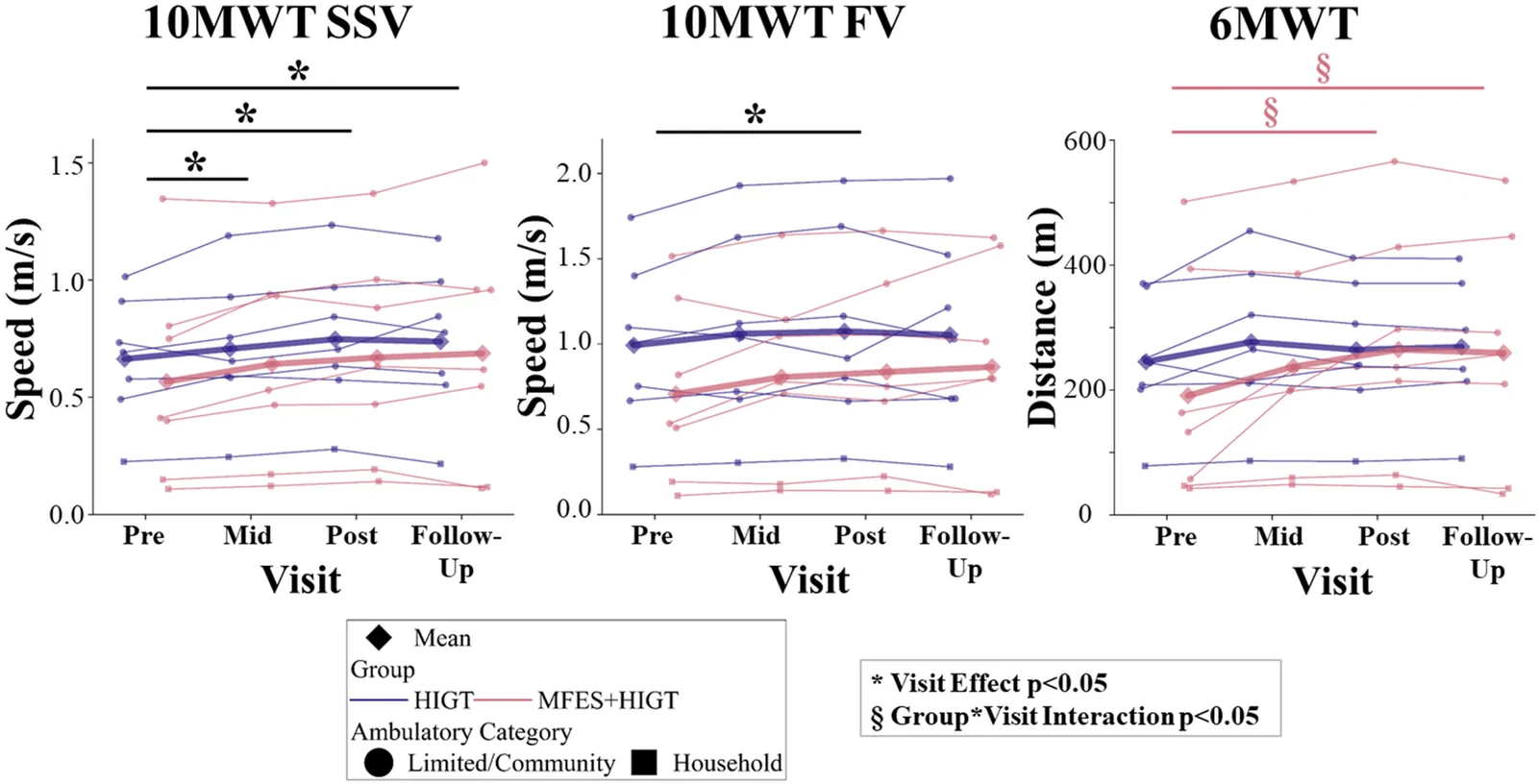
Clinical assessments. 10 Meter Walk Test (10MWT) at self-selected velocity (SSV) and fast velocity (FV) and 6 Minute Walk Test (6MWT) are presented. Improvements for all metrics are positive. Individual data points for each participant are displayed with household ambulators as squares and limited and community ambulators as circles. Significant effects of visit are depicted by black lines and * where *p* < 0.05. Significant group*visit interactions are depicted by lines matching the color of the group associated with the interaction and § where *p* < 0.05. Significant interactions are only observed across visits within group.

Results of clinical assessments are displayed in Figure 3 and Supplementary Table S1. For the 10MWT SSV, there was an effect of visit (*p* = 0.001) but no effect of group (*p* = 0.705) nor interaction (*p* = 0.735). *Post-hoc* analysis of visit revealed, averaged across group, there were improvements at midpoint (*p* = 0.048), post (*p* = 0.001), and follow-up (*p* = 0.005) compared to pre. Two out of seven participants achieved improvements greater than minimal clinically important difference [MCID, 0.14 m/s (64)] in the MFES + HIGT group and only one in the HIGT group. Similarly, the 10MWT FV exhibited an effect of visit (*p* = 0.021) but no effect of group (*p* = 0.392) nor interaction (*p* = 0.638). *Post-hoc* analysis of visit revealed, averaged across group, there was an improvement at post compared to pre (*p* = 0.015). For the 6MWT, there was an effect of visit (*p* = 0.011) and an interaction (*p* = 0.026), but no effect of group (*p* = 0.990). *Post-hoc* analysis of the interaction revealed that, in the MFES + HIGT group there was an improvement from pre (191.3 ± 183.8 m) to post (264.7 ± 187.4 m, *p* = 0.002) and pre to follow-up (259.5 ± 188.2 m, *p* = 0.009), while the HIGT group did not show any improvement (all *p* > 0.444, Supplementary Table S1). No difference between groups at a time point were detected. Four out of seven participants reached MCID of the 6MWT [50 m (64)] in the MFES + HIGT group while only one achieved MCID in the HIGT group. Finally, average number of steps per day did not exhibit any effects (all *p* > 0.406, Supplementary Table S1).

**Figure 4:**
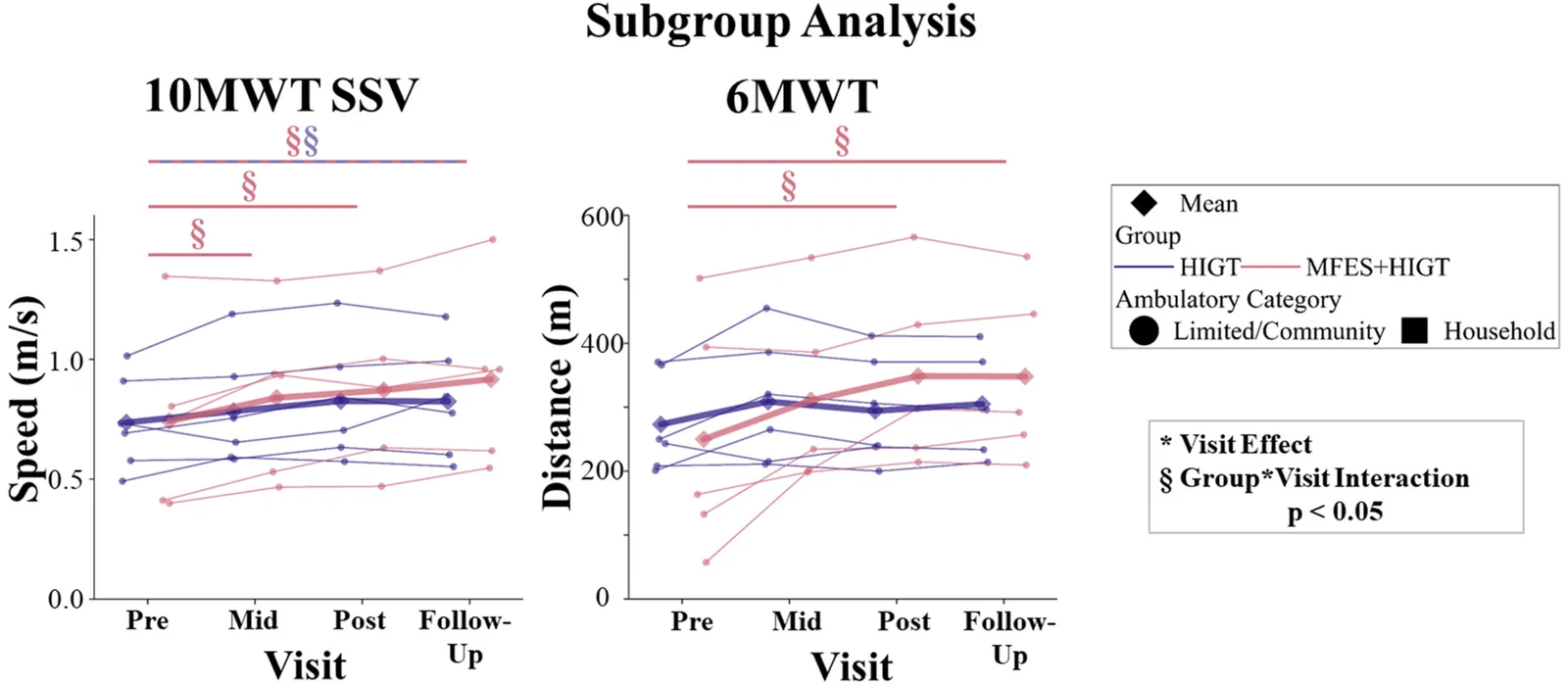
Clinical assessments excluding household ambulators. The 10 Meter Walk Test (10MWT) at self-selected velocity (SSV) and the 6 Minute Walk Test (6MWT) are presented. Individual data points for each participant are displayed, excluding household ambulators. Significant effects of visit are depicted by black lines and * where *p* < 0.05. Significant group*visit interactions are depicted by lines matching the color of the group associated with the interaction and § where *p* < 0.05.

When excluding the household ambulators (Figure 4), 10MWT SSV had an effect of visit (*p* < 0.001) and an interaction (*p* = 0.028), but no effect of group (*p* = 0.849). *Post-hoc* analysis of the interaction revealed that the HIGT group only improved from pre (0.74 ± 0.20 m/s) to follow-up (0.82 ± 0.24 m/s, *p* = 0.017) while the MFES + HIGT group improved from pre (0.74 ± 0.39 m/s) to midpoint (0.84 ± 0.35 m/s, *p* = 0.028), post (0.87 ± 0.35 m/s, *p* = 0.003), and follow-up (0.92 ± 0.38 m/s, *p* < 0.001). The 10MWT FV had an effect of visit (*p* = 0.009), but no effect of group (*p* = 0.681) nor an interaction (*p* = 0.122). *Post-hoc* analysis of visit revealed that, averaged across group, there were improvements from pre to midpoint (*p* = 0.029), post (*p* = 0.011), and follow-up (*p* = 0.042). Analysis of the 6MWT revealed an effect of visit (*p* = 0.003) and an interaction (*p* = 0.008), but no effect of group (*p* = 0.542). *Post-hoc* analysis of the interaction revealed that the MFES + HIGT group improved from pre (250.0 ± 188.6 m) to post (348.8 ± 147.4 m, *p* < 0.001) and follow-up (348.0 ± 137.2 m, *p* = 0.002), while the HIGT group did not show any improvement (all *p* > 0.289). Average steps per day did not achieve significance (*p* > 0.307).

The individual that was a limited community ambulator at baseline had a 6MWT of 58 m despite the limited gait categorization based on walking speed due to fatigue. This individual, however, improved greater than MCID [240m > 50 m (64)] from pre to post, highlighting that MFES + HIGT directly improved endurance, although this individual also improved SSV greater than MCID [0.25 m/s > 0.14 m/s (64)].

#### 3.3.2 Gait biomechanics

**Figure 5:**
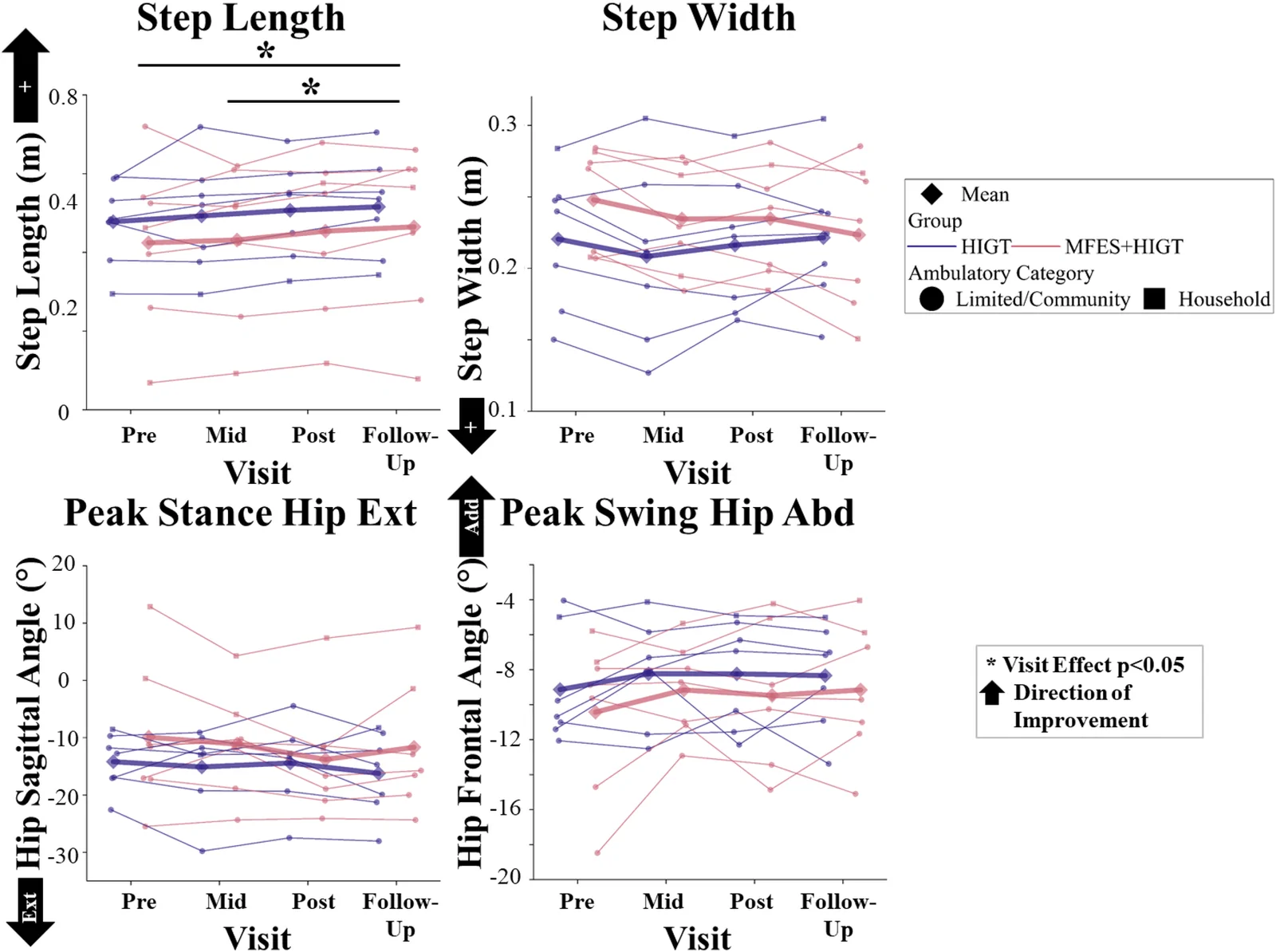
Biomechanics. Top panel: impaired limb step length and step width. Bottom panel: peak impaired limb hip extension angle during stance, peak impaired limb hip abduction (Abd) angle during swing. Extension (Ext) for hip sagittal angle is negative. Adduction (add) for hip frontal angle is positive. Arrows are displayed to depict the direction of improvement. Individual data points for each participant are displayed with household ambulators as squares and limited and community ambulators as circles. Significant effects of visit are depicted by black lines and * where *p* < 0.05.

Selected biomechanics are displayed in Figure 5, all data is displayed in Supplementary Table S1. Impaired limb step length exhibited an effect of visit (*p* = 0.007), while group (*p* = 0.434) and interaction (*p* = 0.922) did not reach significance. Averaged across group, an improvement (increase) was detected from pre to follow-up (*p* = 0.045). Similarly, step width exhibited an effect of visit (*p* = 0.033), while group (*p* = 0.414) and the interaction (*p* = 0.110) failed to reach significance. However, *post-hoc* analysis did not detect any significant contrasts. Neither impaired limb single support nor double support exhibited effects of group, visit, nor interaction (all *p* > 0.099).

Ankle dorsiflexion during swing, knee extension during stance, knee flexion during swing, and hip abduction during swing also did not exhibit any effects of group, visit, nor interactions (all *p* > 0.055). Hip extension during stance phase exhibited an effect of visit (*p* = 0.040), but no contrasts of visit achieved significance (*p* > 0.088) nor did the effect of group (*p* = 0.479) and interaction (*p* = 0.216). In general, there was a modest trend toward increased extension.

When excluding household ambulators, there was an effect of visit for step length (*p* = 0.036), single support (*p* = 0.006), ankle dorsiflexion during swing (*p* = 0.018), and hip extension during stance (*p* = 0.006). *Post-hoc* tests revealed that ankle dorsiflexion was reduced at post compared to pre across groups (*p* = 0.018) and single support increased from pre to follow-up (*p* = 0.022). However, there was no significance across visits on step length and hip extension during stance (*p* > 0.070). There were no other effects nor interactions (*p* > 0.061).

Individual investigation identified some heterogeneous improvements in specific participants. For example, one outlier in the MFES + HIGT group who exhibited the greatest improvement in 6WMT also demonstrated reduced hip abduction—an indicator of reduced hip circumductory gait—and greater hip extension, which has been linked to and may explain improved endurance (pre to post = 240 m). This individual had impaired synergies related to weight acceptance and foot clearance at baseline, as well as high levels of hip abduction during swing phase, suggesting circumductory gait. Greater stability during weight acceptance may have facilitated improved hip extension, and the reduced circumductory gait suggests less compensation for impaired foot clearance although no change in ankle angle was detected. Additionally, this individual exhibited decreased step width at follow-up, which further supports the observed improvement in stability.

To contrast, a higher functioning individual at baseline had impaired synergies to propulsion and foot clearance, and this individual exhibited an improvement in 6MWT above MCID from pre to post (64 m). This individual exhibited an increase in ankle dorsiflexion and reduction in hip abduction during swing, suggesting foot clearance without circumductory gait.

#### 3.3.3 Muscle synergies

**Figure 6:**
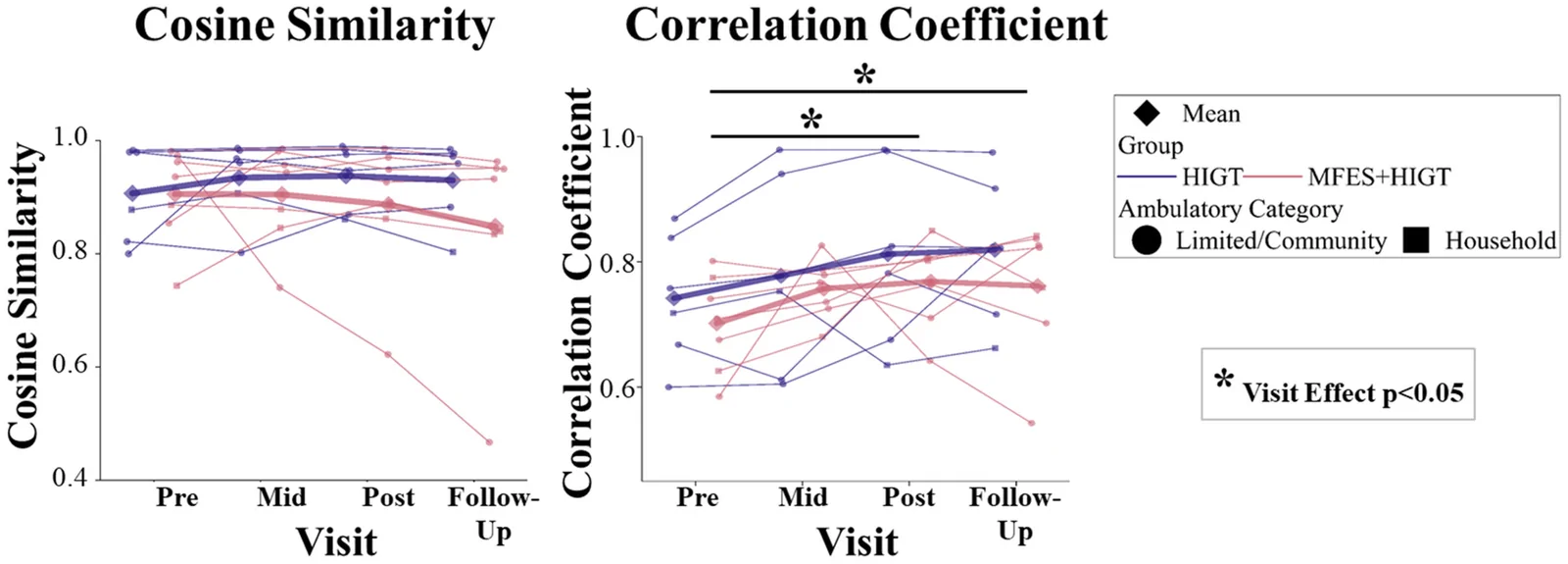
Muscle synergy metrics of impaired synergies at baseline. Cosine similarity and circular cross correlation coefficient were computed between participant synergies that were impaired at baseline and average normative data. A value of one represents synergies that perfectly match the average normative data. Individual data points for each participant are displayed with household ambulators as squares and limited and community ambulators as circles. Significant visit effects are depicted by black lines. * (*p* < 0.05).

On average, four muscle synergies were extracted from neurologically intact individuals, and therefore, four synergies were used for average normative data. Muscle synergies of participants were compared with average normative data and results are displayed in Figure 6 and Supplementary Table S1. On average, 2.1 ± 1.6 and 2.3 ± 0.8 synergies were impaired at baseline for the HIGT and MFES + HIGT group, respectively. In both groups, the synergy related to propulsion was most frequently impaired (HIGT: 5/7, MFES + HIGT: 6/7) while the synergy related to weight acceptance was least frequently impaired (2/7 in both groups).

Cosine similarity of weights had no effect of group (*p* = 0.253), visit (*p* = 0.244), nor interaction (*p* = 0.427). Circular cross correlation coefficient of activation profile had an effect of visit (*p* = 0.005) but no effect of group (*p* = 0.473) nor interaction (*p* = 0.850). *Post-hoc* analysis revealed that, averaged across group, correlation coefficient improved from pre to post (*p* = 0.024) and pre to follow-up (*p* = 0.049). The number of components extracted from NNMF had no effect of group (*p* = 0.150), visit (*p* = 0.095), nor interaction (*p* = 0.771). The synergies related to knee flexion (HIGT: 4/7, MFES + HIGT: 3/7) and foot clearance (HIGT: 4/7, MFES + HIGT: 5/7) were also frequently impaired. When excluding household ambulators, no neuromuscular outcomes exhibited any effects of group, visit, nor interactions (*p* > 0.132). However, individual investigation did identify some heterogeneous improvements in specific participants. For example, the outlier in the MFES + HIGT group who exhibited the greatest improvement in 6WMT surprisingly demonstrated the greatest reduction in cosine similarity of synergies. This may suggest that there was a change in the structure of the weight acceptance and foot clearance synergies to achieve this improved endurance, but the change in structure did not increase the similarity to normative data. In contrast, the higher level participant considered in the previous section did not exhibit any major changes in synergy structure or timing.

### 3.4 Clinical feasibility

**Figure 7:**
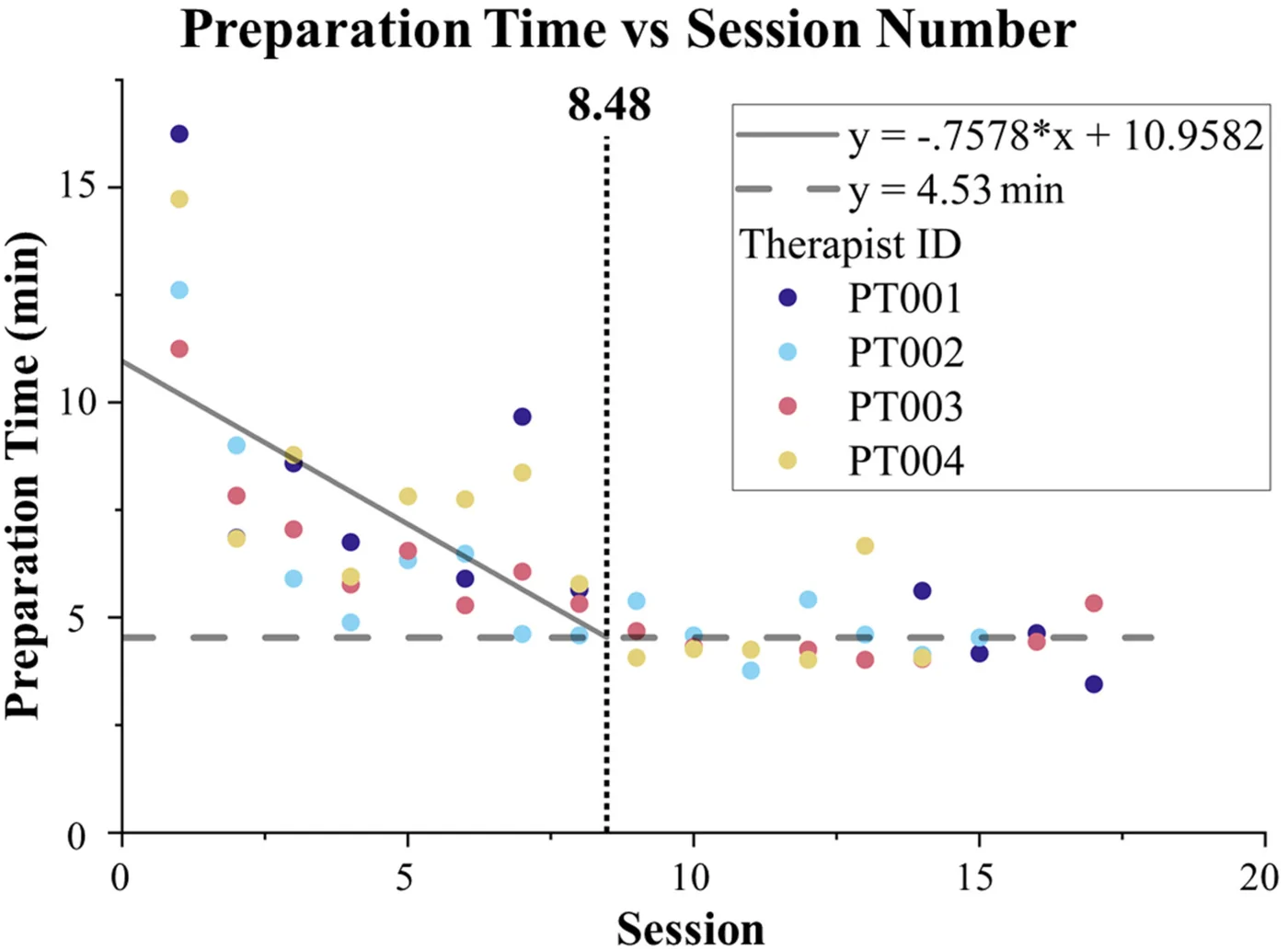
Preparation time vs. Number of Sessions. The time to don the sleeve and set parameters was recorded for each session for each of the four therapists, who are each depicted with a different color as specified in the legend. An inflection point analysis was conducted to identify the learning region and the plateau region.

System setup time for the four therapists was recorded with four of the seven participants in the MFES + HIGT group and assessed for feasibility of clinical implementation (Figure 7). Each data point represents a single session with a single participant for each therapist. Setup time decreased across sessions and was well described by a linear–plateau model, which identified an inflection point at 8.48 sessions with an estimated plateau setup time of 4.53 min. Therapists also completed the Therapist Perceived Usefulness Questionnaire on whether they would implement synergy-based MFES + HIGT into their own clinical practice and reported a mean score of +3.6 ± 0.48. Therapist feedback highlighted ease of use to don the sleeve and update stimulation parameters throughout training. For the Implementation Outcome Measures (61), ranging from 1 to 5 and assess feasibility, appropriateness, and acceptability of intervention, therapists reported an average score of 3.9 ± 0.13, 4.1 ± 0.13, and 4.0 ± 0.00, respectively. Lastly, on the 10-item System Usability Scale, average therapist score for synergy-based MFES was 72.15 ± 7.5 indicating an acceptable rating (65).

We also collected participant feedback at follow-up with the Patient Perceived Usefulness Questionnaire. On average, the MFES + HIGT group rating was +3.43 ± 1.51, while the HIGT group rating was +3.14 ± 0.38, which were not significantly different (*p* = 0.636). Qualitative feedback from both groups highlighted improved endurance, but notably, multiple participants in the MFES + HIGT group emphasized reduced reliance on assistive devices.

## 4 Discussion

In this pilot study, we investigated preliminary implementation of personalized synergy-based MFES with HIGT for chronic stroke gait rehabilitation and compared efficacy with HIGT. Both groups on average achieved target HR zones outlined for HIGT in clinical practice guidelines (5), but household ambulators (*N* = 3, baseline 10MWT SSV < 0.4 m/s) failed to maintain target HR. Despite the small sample size and non-significant but greater baseline function in the HIGT group, only the MFES + HIGT group demonstrated a significant improvement in endurance–although no difference between groups was detected at any time point. Both groups moderately improved walking speed, impaired limb step length, and similarity of synergy timings to controls. In an exploratory analysis, household ambulators were removed, as these participants did not achieve HIGT targets. In this subset, endurance and SSV improved only in the MFES + HIGT group from pre to post, highlighting the need for future studies to systematically investigate how baseline function impacts effectiveness of synergy-based MFES. Additionally, physical therapists became proficient in preparing MFES sessions, plateauing in setup time after nine sessions at 4.53 min, which we believe to be feasible for clinical implementation. Furthermore, therapists rated the feasibility, appropriateness, acceptability, and usability of the intervention highly.

### 4.1 Training intensity

On average, each group was able to achieve target HR zones (70%–85%) for 50% of each session with no dropouts in the MFES + HIGT group (one dropout in the HIGT group due to a preexisting medical condition). This was consistent with prior studies (7), where participants maintained target HR zones for at least 30% of a training session. However, the three household ambulators on average reached target HR zones 22.9 ± 14.3% of the time. This is potentially due to the limited ability to increase speed and incline for these participants, thereby limiting our ability to effectively challenge them and elevate their heart rate. This finding likely reflects limitations in overall mechanical capacity rather than inadequate muscle activation alone. Because synergy-based MFES is intended to augment neuromuscular coordination rather than provide substantial propulsive assistance, additional technologies that reduce the mechanical demands of walking (e.g., body-weight support or robotic gait assistance) may help these individuals achieve the cardiovascular training intensity recommended for HIGT while preserving the neuromuscular benefits of personalized stimulation (5, 66). However, these participants did still rate the intervention as challenging using the RPE scale. Therefore, this intervention may be more appropriate for moderate and higher-functioning individuals.

### 4.2 Training efficacy

The present study found that while both groups demonstrated improvements in walking speed, impaired limb step length, and similarity of muscle synergy activation profiles to normative data, only the MFES + HIGT group achieved significant and sustained gains in walking endurance. However, no difference in improvement between groups was detected. Due to small the sample size, we cannot conclude that MFES + HIGT promoted better improvements in endurance than HIGT, but these results support further investigation in a higher-powered study to determine if synergy-based MFES outperforms HIGT. We did find that four out of seven participants in the MFES + HIGT group surpassed the MCID threshold for 6MWT compared to one in the HIGT group. Additionally, to provide context to these improvements, Springer et al. assessed improvements in gait with FES to the TA and either hamstring or quadriceps (67). They observed after six weeks of training a mean increase in speed during the 2-minute walk test of 0.08 m/s during the test in the dual channel FES group while participants undergoing synergy-based MFES in this study improved their speed from pre to post during the 6MWT by 0.20 m/s. Endurance remains a critical challenge in stroke rehabilitation (4), and greater walking endurance has been linked to better community reintegration outcomes (68). Our findings are consistent with prior studies showing that extending FES to both the TA and gluteus medius can improve endurance in individuals with chronic stroke (22, 23), with some groups showing improvement in both the MFES and control groups without a significant between group difference (24, 69), highlighting the need to further investigate responders to this treatment. Although, it is worth noting that in these studies the average 6MWT distance at baseline of the MFES group was 302 ± 37 m (24) and 530 ± 262 m (69), while the baseline 6MWT distance at baseline of the MFES group in the present study was 191.6 ± 183.6 m and the algorithms were not personalized to each individual. Similarly, FastFES, which combines FES to the TA and GAS, has been shown to reduce the metabolic cost of walking (24, 25), although this was not investigated in this study.

We hypothesize that these endurance gains may be due to both peripheral (10) and central mechanisms (11–15). Peripherally, FES may have improved propulsive force by enhancing activation of the gastrocnemius, a key muscle in the propulsive muscle synergy that was impaired in eleven out of fourteen participants at baseline. Although not directly measured, propulsion of the impaired limb is an essential predictor of 6MWT distance (70). Repetitive, precisely timed FES may have increased muscle mass and strength (71, 72), facilitating more efficient energy expenditure, as shown in previous FastFES studies (24, 25). Centrally, we observed that muscle synergy activation profiles became more similar to normative data in both groups and accompanied improved gait speed and endurance with training, consistent with previous work demonstrating that gait rehabilitation improves both synergies and gait outcomes (31–33). Previous research has shown that FES can promote neuroplasticity and reshape neural coordination (16, 17), and muscle synergy-based stimulation technique has also been shown to reduce FES-induced fatigue in the upper limb compared to other stimulation strategies (73).

While spatial organization of synergies (i.e., cosine similarity) did not exhibit a significant change in either group, one participant in the MFES + HIGT group dramatically reduced cosine similarity, becoming more dissimilar to normative data with no change in correlation coefficient. However, they also achieved the greatest improvements in walking speed (SSV_Post−Pre_=0.25 m/s) and distance (6MWT_Post−Pre_=240 m), as well as reduced hip abduction during swing by 5° (greatest among all participants) and increased hip extension at stance by 3.5° from pre to post, suggesting reduced compensatory movements and better propulsion. Therefore, muscle synergy organization was modified with training, but not in a way we would expect, yet it still produced substantial functional improvement without compensatory biomechanics. As this is a single case, further research is required in a larger population to characterize how muscle synergy patterns change as a result of synergy-based MFES training and investigate alternative changes in synergy organization that still minimize compensatory mechanisms.

### 4.3 Subgroup analysis

Household ambulators did not achieve target HR zones and thus did not effectively receive the full benefits of either intervention. Therefore, we conducted an exploratory subgroup analysis investigating changes in mild-to-moderate impaired individuals [SSV > 0.4 m/s (51)]. Ankle dorsiflexion during swing was reduced from pre to post when pooling across groups, with MFES + HIGT resulting in a 2° reduction and HIGT resulting in a 4° reduction in dorsiflexion angle during swing, but no between group differences were detected. It is also worth noting that baseline ankle dorsiflexion angles during swing were minimal to begin with, but future efforts need to be made to improve ankle mechanics. No changes in hip circumduction were detected, suggesting that this change did not incur any additional strain on the body. Muscle synergy metrics were investigated, but no changes from baseline compared to the full dataset were detected. 6MWT still improved for the MFES + HIGT from pre to post and follow-up while no changes were observed in the HIGT group. To extend the comparison with the study by Springer et al., the change in gait speed during the 6MWT from pre to post in this subgroup was 0.27 m/s.

Additionally, subgroup analysis detected that only the MFES + HIGT group improved SSV from pre to midpoint and post, while both groups improved from pre to follow-up. From these results, we cautiously conclude that synergy-based MFES + HIGT has potential to improve endurance and gait speed in individuals with mild-to-moderate impairment.

Furthermore, synergy-based MFES + HIGT may be most appropriate for individuals with mild and moderate impairment because they can maintain target HR zones of HIGT to receive the full benefits of the intervention. This highlights the importance of selecting the target population for future randomized controlled trials. Household ambulators (SSV < 0.4 m/s) generally did not achieve the prescribed high-intensity training and showed limited response to either intervention, suggesting that this population may require modified training protocols or different interventions. One strategy to enhance the ability of more severely impaired individuals to reach higher intensities is to combine synergy-based MFES with soft robotics, which may further enhance muscle coordination and joint kinematics with training (74–76). We believe that this preliminary work supports further investigation into the feasibility in a larger population of therapists with greater diversity of experience as well as a randomized controlled trial to better characterize improvements achieved from synergy-based MFES and identify optimal responders and non-responders based on baseline function.

Our findings underscore a fundamental tension in clinical research: while personalization is essential for effective rehabilitation, it can also conflict with the statistical power required in randomized controlled trials. Broad inclusion criteria risk masking treatment effects due to high inter-individual variability, whereas overly restrictive criteria carry risk of excluding those who may benefit. Therefore, pilot studies, such as the one presented here, are critical for refining inclusion and exclusion criteria, helping to identify the most appropriate target population for future randomized controlled trials. Ultimately, determining this balance is a critical step toward advancing personalized, evidence-based interventions.

### 4.4 Clinical feasibility

The MFES + HIGT intervention was well-received and tolerated by both participants and therapists, with MFES + HIGT participants qualitatively reporting improved confidence, balance, and independence from assistive devices. Setup time decreased with practice across sessions. A linear–plateau model explained a moderate proportion of variance (R² = 0.59) and identified an inflection point at 8.48 sessions, after which setup time stabilized at 4.53 min. A study investigating currently used rehabilitation technologies in the clinical setting found that average setup time was 2.9 ± 4.3 min, with 84% of technologies taking less than five minutes (9). Therefore, this intervention appears to be feasible for clinical implementation. Since setup time was collected with one individual post-stroke for each therapist, decreased setup time may be attributed to both the therapist learning and reduced system adjustment over time with the same person. Additionally, the System Usability Scale indicated acceptable usability with an average of 71.5, passing the threshold for acceptable usability of 68/100 (65, 77). In addition, the Perceived Usefulness questionnaire indicated that therapists were likely to implement the intervention into their clinical practice, while technical support is preferred. However, manual placement of the custom external hip unit led to greater setup times, reduced confidence when manually placing electrodes, and a few disconnects between the system and the external unit, which is when technical support was necessary.

### 4.5 Limitations and future directions

This is a pilot study that was limited by a small sample size and therefore not powered to identify between group differences, limiting the generalizability of these results. Furthermore, the baseline function, time since stroke, and age of the participants were quite variable with the HIGT group exhibiting slightly greater baseline function, although the difference was not significant. These limitations taken together potentially limited the statistical power and ability to achieve significance between group improvements. This study did not include blinded assessors, and only individuals in the MFES + HIGT group wore the sleeve during training, potentially leading to confounding effects of improvements due to compression. These limitations will need to be addressed in a future powered randomized controlled trial. Future studies should also include metabolic cost so the intervention can be compared to FastFES (18, 24, 25), comparison to other approaches to FES (e.g., different number of muscles, different approach to personalization, generalized approach), and investigate different strategies to construct personalized MFES interventions targeting both limbs as stroke indeed impairs both limbs [as was done in our pilot study (39)]. Additionally, biomechanics were not assessed during each training session but acute changes with FES should be considered in future studies. Also, while electrode placement was consistent, the size of the electrodes could lead to activation of neighboring muscles in addition to the target muscle, but individual muscle testing by therapists ensured that stimulation of each muscle activated the proper biomechanical response. A sensitivity analysis on placement of the hip unit would be necessary to validate consistency of its placement, although incorporation into the sleeve would also improve placement consistency. Additionally, selecting a VAF threshold of 0.9 to determine the number of synergies in neurologically intact individuals for this study may have impacted the design of the FES waveforms. However, literature has consistently explained neurologically intact gait with four synergies with the biomechanical functions that were identified in this study, reinforcing the physiological basis of the selection (26, 29, 30).

In the future, smaller electrodes could be used on the sleeve to ensure very precise activation. Combining synergy-based MFES with soft robotics in a hybrid solution may further enhance muscle coordination and joint kinematics with training (74–76), while recent advancements in real time extraction of synergies and operant conditioning via visual feedback offer another avenue for personalized interventions (78–83). For clinical translation, ongoing assessment of product usability should be considered to keep setup time acceptable within the clinical workflow.

## 5 Conclusion

In a small, variable sample of individuals living with lasting gait impairments due to chronic stroke, a personalized stimulation approach–muscle synergy-based multichannel functional electrical stimulation–paired with high-intensity gait training demonstrated preliminary improvement in endurance and was feasibly implemented in the clinical setting. Furthermore, despite the small sample size, baseline function appears to impact recovery potential, which could be used to inform inclusion/exclusion criteria for future studies. We posit that these preliminary results support pursuit of a larger, randomized controlled trial that can more effectively compare improvements between groups and identify responders based on baseline function. Leveraging approaches such as synergy-based MFES, which precisely tailors a rehabilitation intervention to an individual's impaired neuromuscular coordination, holds great promise for translating personalized rehabilitation into clinical practice and enhancing recovery.

## Data Availability

The raw data supporting the conclusions of this article will be made available by the authors, without undue reservation.
