# Supplementary material for: Synergy-based Functional Electrical Stimulation for gait rehabilitation in chronic stroke: a pilot study
Source: Front Rehabil Sci. 2026 Sep 9;7:1847719. doi: 10.3389/fresc.2026.1847719 (PMC13597845; doi:10.3389/fresc.2026.1847719)
Supplement: Supplementary file 1 [file Datasheet1.docx]

Supplementary Material

# 1 Supplementary Figures and Tables

For more information on Supplementary Material and for details on the different file types accepted, please see [here](https://www.frontiersin.org/guidelines/author-guidelines#supplementary-material).

## 1.1 Supplementary Figures

**Supplementary Figure 1.** A. 1) Steps for Personalized Stimulation. Starting from EMG recorded during baseline assessment for each stroke participant, synergies are extracted using an interative nonnegative matrix factorization algorithm. These synergies are compared to normative synergies extracted from neurologically intact individuals. 2) Based on cosine similarity and circular cross correlation comparisons of each synergy to its normative synergy counterpart, 3) if a synergy exceeded two standard deviations away from the normative synergy. In this example, the weight acceptance synergy is not impaired because it meets the similarity criteria whereas the other three synergies are impaired. 4) Nonnegative matrix reconstruction of each muscle is computed with only the synergies identified as impaired. Then, stimulation profiles are normalized to maximum value for each muscle and thresholded at 0.2, such that any value below 0.2 is set to zero. This is done to ensure that stimulation is not active throughout the entire gait cycle, which would induce fatigue more rapidly. 5) Stimulation profiles were projected on 100% of the gait cycle. Stimulation is triggered based on six IMU events identified as sagittal shank and thigh peaks and zero crossings. IMU events were selected with consistent timing throughout the gait cycle from neurologically intact individuals. In real time, the duration of the gait cycle is updated with an exponential moving average from heel strike to heel strike. Stimulation onset and offset for each phase were triggered by the corresponding IMU event occurring in real time during walking, with brief delays applied when necessary to align stimulation with the prescribed timing profile. B) Example stimulation profiles for two individuals with different synergy profiles, with profiles shown for each muscle.

## 1.2 Supplementary Tables

Supplemental Table I Intervention Efficacy. * p<0.05 for visit effects. § p<0.05 for group*visit interactions. The arrows indicate the direction of improvement. 10MWT = 10 Meter Walk Test, SSV = Self-Selected Velocity, FV = Fast Velocity, 6MWT = 6 Minute Walk Test

| Outcome | Group | Time Point | | | | Linear Mixed Model P-Value | | |
| --- | --- | --- | --- | --- | --- | --- | --- | --- |
|  |  | Pre | Mid | Post | Follow-Up | Group | Visit | Group*Visit |
| Clinical | | | | | | | | |
| 10MWT SSV (m/s) ↑ | MFES+HIGT | 0.57±0.43 | 0.64±0.44 | 0.70±0.45 | 0.69±0.50 | 0.705 | 0.001* | 0.735 |
|  | HIGT | 0.66±0.26 | 0.71±0.30 | 0.75±0.31 | 0.74±0.32 |  |  |  |
| 10MWT FV (m/s) ↑ | MFES+HIGT | 0.71±0.53 | 0.81±0.53 | 0.84±0.56 | 0.87±0.61 | 0.392 | 0.021* | 0.638 |
|  | HIGT | 0.99±0.48 | 1.06±0.56 | 1.07±0.58 | 1.05±0.57 |  |  |  |
| 6MWT (m) ↑ | MFES+HIGT | 191.3±183.8 | 237.4±173.3 | 264.7±187.4 | 259.5±188.2 | 0.990 | 0.011* | 0.026 § |
|  | HIGT | 245.4±101.2 | 277.0±122.5 | 264.6±109.6 | 269.1±116.0 |  |  |  |
| Average Steps Per Day ↑ | MFES+HIGT | 2777±2089 |  | 2901±2007 |  | 0.406 | 0.701 | 0.991 |
|  | HIGT | 2040±1586 |  | 1904±1037 |  |  |  |  |
| Biomechanics | | | | | | | | |
| Step Length (m) ↑ | MFES+HIGT | 0.42±0.21 | 0.43±0.20 | 0.45±0.20 | 0.46±0.21 | 0.434 | 0.007* | 0.922 |
|  | HIGT | 0.48±0.11 | 0.49±0.14 | 0.51±0.12 | 0.52±0.13 |  |  |  |
| Step Width (mm)↓ | MFES+HIGT | 0.25±0.04 | 0.23±0.04 | 0.23±0.04 | 0.22±0.05 | 0.414 | 0.033* | 0.110 |
|  | HIGT | 0.22±0.05 | 0.21±0.06 | 0.22±0.05 | 0.22±0.05 |  |  |  |
| Single Support (%) ↑ | MFES+HIGT | 28.8±5.2 | 28.5±4.9 | 29.0±5.5 | 28.8±6.1 | 0.379 | 0.212 | 0.561 |
|  | HIGT | 31.1±3.9 | 31.1±4.3 | 31.1±4.4 | 31.7±5.1 |  |  |  |
| Double Support (%) ↓ | MFES+HIGT | 40.3±9.1 | 39.9±8.8 | 37.9±8.3 | 39.7±10.5 | 0.371 | 0.121 | 0.099 |
|  | HIGT | 36.4±6.5 | 35.4±6.9 | 35.7±6.3 | 35.9±7.8 |  |  |  |
| Ankle Dorsiflexion in Swing (°) ↑ | MFES+HIGT | -8.6±3.6 | -8.8±4.6 | -10.2±3.4 | -9.2±3.1 | 0.117 | 0.055 | 0.377 |
|  | HIGT | -4.4±5.9 | -7.3±4.5 | -8.0±4.3 | -.6.0±2.5 |  |  |  |
| Knee Extension in Stance (°) ↑ | MFES+HIGT | -5.2±6.5 | -5.5±5.1 | -2.5±3.9 | -2.5±2.5 | 0.625 | 0.889 | 0.139 |
|  | HIGT | -3.7±4.6 | -3.0±4.4 | 2.3±3.7 | -2.8±5.1 |  |  |  |
| Knee Flexion in Swing (°) ↓ | MFES+HIGT | -0.53±0.31 | -0.56±0.28 | -0.58±0.31 | -0.56±0.26 | 0.576 | 0.854 | 0.536 |
|  | HIGT | -0.65±0.29 | -0.66±0.32 | -0.65±0.32 | -0.63±0.34 |  |  |  |
| Hip Extension in Stance (°) ↓ | MFES+HIGT | -9.9±12.8 | -11.1±9.1 | -13.8±10.4 | -11.7±11.7 | 0.479 | 0.040* | 0.216 |
|  | HIGT | -14.2±4.9 | -15.1±7.3 | -14.4±7.3 | -16.2±7.2 |  |  |  |
| Hip Abduction in Swing (°) ↑ | MFES+HIGT | -10.4±4.5 | -9.2±2.7 | -9.5±3.9 | -9.2±3.8 | 0.509 | 0.237 | 0.948 |
|  | HIGT | -9.1±3.2 | -8.2±3.0 | -8.2±3.1 | -8.3±3.0 |  |  |  |
| Muscle Synergies | | | | | | | | |
| Cosine Similarity ↑ | MFES+HIGT | 0.91±0.09 | 0.90±0.09 | 0.90±0.12 | 0.85±0.18 | 0.253 | 0.244 | 0.427 |
|  | HIGT | 0.91±0.08 | 0.93±0.07 | 0.94±0.06 | 0.93±0.07 |  |  |  |
| Cross Corr. Coefficient ↑ | MFES+HIGT | 0.70±0.07 | 0.76±0.05 | 0.77±0.07 | 0.76±0.10 | 0.473 | 0.005* | 0.850 |
|  | HIGT | 0.74±0.10 | 0.78±0.16 | 0.81±0.15 | 0.82±0.12 |  |  |  |
| Number of Synergies ↑ | MFES+HIGT | 3.0±0.6 | 2.9±0.7 | 3.1±0.9 | 3.1±0.7 | 0.767 | 0.766 | 1.000 |
|  | HIGT | 3.3±0.8 | 3.1±0.9 | 3.7±0.5 | 3.7±0.8 |  |  |  |

Supplemental Table II Sub-group Analysis of Clinical Assessments. * p<0.05 for visit effects. § p<0.05 for group*visit interactions. The arrows indicate the direction of improvement. 10MWT = 10 Meter Walk Test, SSV = Self-Selected Velocity, FV = Fast Velocity, 6MWT = 6 Minute Walk Test.

| Outcome | Group | | Time Point | | | | | | | Linear Mixed Model P-Value | | | |
| --- | --- | --- | --- | --- | --- | --- | --- | --- | --- | --- | --- | --- | --- |
|  |  |  | Pre | Mid | | Post | | Follow-Up | | Group | Visit | | Group*Visit |
| Clinical | | | | | | | | | | | | | |
| 10MWT SSV (m/s) ↑ | MFES+HIGT | | 0.74±0.39 | 0.84±0.35 | | 0.87±0.35 | | 0.92±0.38 | | 0.849 | <0.001* | | 0.028 § |
|  | HIGT | | 0.74±0.20 | 0.78±0.24 | | 0.83±0.25 | | 0.82±0.24 | |  |  |  |  |
| 10MWT FV (m/s) ↑ | MFES+HIGT | | 0.93±0.45 | 1.06±0.37 | | 1.10±0.42 | | 1.16±0.41 | | 0.681 | 0.009* | | 0.122 |
|  | HIGT | | 1.11±0.40 | 1.19±0.50 | | 1.20±0.52 | | 1.18±0.50 | |  |  |  |  |
| 6MWT (m) ↑ | MFES+HIGT | | 250.0±188.6 | 310.8±146.6 | | 348.8±147.4 | | 348.0±137.2 | | 0.542 | 0.003* | | 0.008 § |
|  | HIGT | | 273.2±76.1 | 308.8±97.6 | | 294.4±83.4 | | 304.9±85.0 | |  |  |  |  |
| Average Steps Per Day ↑ | MFES+HIGT | | 3409±2189 |  | | 3343±1888 | |  | | 0.307 | 0.619 | | 0.811 |
|  | HIGT | | 2173±1735 |  | | 2023±1083 | |  | |  |  |  |  |
| Biomechanics | | | | | | | | | | | | | |
| Step Length (m) ↑ | | MFES+HIGT | 0.49±0.17 | | 0.48±0.16 | | 0.50±0.17 | | 0.52±0.16 | 0.622 | | 0.036* | 0.441 |
|  |  | HIGT | 0.51±0.08 | | 0.53±0.12 | | 0.54±0.10 | | 0.54±0.11 |  |  |  |  |
| Step Width (mm)↓ | | MFES+HIGT | 0.25±0.04 | | 0.24±0.04 | | 0.24±0.04 | | 0.23±0.05 | 0.158 | | 0.061 | 0.515 |
|  |  | HIGT | 0.21±0.04 | | 0.19±0.05 | | 0.20±0.03 | | 0.21±0.03 |  |  |  |  |
| Single Support (%) ↑ | | MFES+HIGT | 31.2±4.0 | | 30.8±3.4 | | 31.7±3.4 | | 32.0±3.1 | 0.353 | | 0.006* | 0.555 |
|  |  | HIGT | 32.5 ±1.0 | | 32.6±1.5 | | 32.6±2.0 | | 33.5±2.4 |  |  |  |  |
| Double Support (%) ↓ | | MFES+HIGT | 34.2±4.4 | | 33.9±4.6 | | 32.6±5.1 | | 33.4±5.1 | 0.589 | | 0.262 | 0.123 |
|  |  | HIGT | 32.5±0.95 | | 31.7±1.7 | | 32.2±2.6 | | 32.3±2.5 |  |  |  |  |
| Ankle Dorsiflexion in Swing (°) ↑ | | MFES+HIGT | -9.1±3.2 | | -9.6±5.2 | | -11.0±3.5 | | -9.2±3.7 | 0.171 | | 0.018* | 0.634 |
|  |  | HIGT | -4.6±6.5 | | -8.0±4.5 | | -8.9±3.9 | | -6.4±2.4 |  |  |  |  |
| Knee Extension in Stance (°) ↑ | | MFES+HIGT | -3.6±4.9 | | -4.6±4.8 | | -1.8±3.6 | | -2.3±2.9 | 0.952 | | 0.114 | 0.469 |
|  |  | HIGT | -4.2±4.77 | | -3.0±4.9 | | -2.2±4.1 | | -3.2±5.4 |  |  |  |  |
| Knee Flexion in Swing (°) ↓ | | MFES+HIGT | -0.63±0.29 | | -0.67±0.23 | | -0.72±0.21 | | -0.68±0.18 | 0.850 | | 0.280 | 0.567 |
|  |  | HIGT | -0.70±0.29 | | -0.72±0.30 | | -0.71±0.30 | | -0.69±0.33 |  |  |  |  |
| Hip Extension in Stance (°) ↓ | | MFES+HIGT | -16.5±5.8 | | -15.2±6.2 | | -18.4±4.8 | | -17.9±4.4 | 0.696 | | 0.006* | 0.079 |
|  |  | HIGT | -15.1±4.7 | | -15.5±7.9 | | -14.7±7.9 | | -17.6±6.9 |  |  |  |  |
| Hip Abduction in Swing (°) ↑ | | MFES+HIGT | -11.9±4.5 | | -10.3±2.0 | | -11.4±2.6 | | -10.3±2.0 | 0.182 | | 0.269 | 0.623 |
|  |  | HIGT | -9.8±2.9 | | -8.9±2.6 | | -8.8±2.6 | | -8.9±2.8 |  |  |  |  |
| Muscle Synergies | | | | | | | | | | | | | |
| Cosine Similarity ↑ | | MFES+HIGT | 0.94±0.05 | | 0.92±0.10 | | 0.89±0.15 | | 0.85±0.22 | 0.520 | | 0.747 | 0.248 |
|  |  | HIGT | 0.91±0.09 | | 0.94±0.08 | | 0.95±0.05 | | 0.96±0.04 |  |  |  |  |
| Cross Corr. Coefficient ↑ | | MFES+HIGT | 0.70±0.08 | | 0.77±0.04 | | 0.75±0.07 | | 0.75±0.13 | 0.454 | | 0.132 | 0.815 |
|  |  | HIGT | 0.75±0.11 | | 0.78±0.18 | | 0.85±0.13 | | 0.85±0.10 |  |  |  |  |
| Number of Synergies ↑ | | MFES+HIGT | 3.0±0.6 | | 2.9±0.7 | | 3.1±0.9 | | 3.2±0.8 | 0.631 | | 0.724 | 0.955 |
|  |  | HIGT | 3.3±0.8 | | 3.1±0.9 | | 3.7±0.5 | | 3.7±0.8 |  |  |  |  |
